# Identification of the transcriptional response of human intestinal mucosa to *Lactobacillus plantarum *WCFS1 *in vivo*

**DOI:** 10.1186/1471-2164-9-374

**Published:** 2008-08-05

**Authors:** Freddy J Troost, Peter van Baarlen, Patrick Lindsey, Andrea Kodde, Willem M de Vos, Michiel Kleerebezem, Robert-Jan M Brummer

**Affiliations:** 1 Dept. of Internal Medicine, div. of Gastroenterology & Hepatology, Maastricht University, Maastricht, The Netherlands; 2 Top Institute Food & Nutrition (TIFN), Wageningen, The Netherlands; 3 Center for Molecular and Biomolecular Informatics, Radboud University Medical Centre Nijmegen, Nijmegen, The Netherlands; 4 Nizo Food Research, Ede, The Netherlands; 5 Department of Population Genetics, Maastricht University, Maastricht, The Netherlands; 6 Dept. of Microbiology, Wageningen University and Research, Wageningen, The Netherlands; 7 Depts. of Internal Medicine and Clinical Dietetics, University Hospital Maastricht, Maastricht, The Netherlands

## Abstract

**Background:**

There is limited knowledge on the extent and dynamics of the mucosal response to commensal and probiotic species in the human intestinal lumen. This study aimed to identify the acute, time-dependent responses of intestinal mucosa to commensal *Lactobacillus plantarum *WCFS1 *in vivo *in two placebo-controlled human intervention studies in healthy volunteers. Transcriptional changes in duodenal mucosa upon continuous intraduodenal infusion of *L. plantarum *WCFS1 for one- and six h, respectively, were studied using oro- and nasogastric intubations with dedicated orogastric catheters and tissue sampling by standard flexible gastroduodenoscopy.

**Results:**

One- and six-h exposure of small intestinal mucosa to *L. plantarum *WCFS1 induced differential expression of 669 and 424 gene reporters, respectively. While short-term exposure to *L. plantarum *WCFS1 inhibited fatty acid metabolism and cell cycle progression, cells switched to a more proliferative phase after prolonged exposure with an overall expression profile characterized by upregulation of genes involved in lipid metabolism, cellular growth and development. Cell death and immune responses were triggered, but cell death-executing genes or inflammatory signals were not expressed. Proteome analysis showed differential expression of several proteins. Only the microsomal protein 'microsomal triglyceride transfer protein' was regulated on both the transcriptional and the protein level in all subjects.

**Conclusion:**

Overall, this study showed that intestinal exposure to *L. plantarum *WCFS1 induced consistent, time-dependent transcriptional responses in healthy intestinal mucosa. This extensive exploration of the human response to *L. plantarum *WCFS1 could eventually provide molecular support for specific or probiotic activity of this strain or species, and exemplifies the strength of the applied technology to identify the potential bio-activity of microbes in the human intestine.

## Background

Many lactic acid bacteria (LAB) have a long history of use in the preservation of food ingredients [1]. In addition to their preservative effect, which is largely based on their effectiveness to convert the available carbon sources in the raw materials to lactic and acetic acid, LAB-fermentation generates many other relevant product characteristics like texture, flavor and stability. Various LAB species, given as food supplements, exert beneficial bioactivity in the intestine [2,3].

The study of interactions between the human host and microbes that confer a health promoting effect is mostly limited to an evaluation of the effects of the microbes on gastrointestinal or systemic symptom scores. Several studies described beneficial effects of dietary supplementation with various LAB, including specific strains of the species *Lactobacillus plantarum *on symptoms in patients suffering from irritable bowel syndrome (IBS) [4], in inflammatory bowel disease (IBD) patients [5], and on the intestinal barrier function in stressed conditions [6]. Detailed, descriptive studies of the human mucosal response to LAB are scarce. DiCaro et al. investigated the effects of *L. rhamnosus *GG on transcriptional responses of small intestinal mucosa in oesophagitis patients [7], delivering a myriad of information about the impact of a probiotic supplementation regimen on the human mucosal response to probiotics in a limited number (n = 3) of subjects suffering from an underlying medical condition.

The present study aimed to identify the response of healthy intestinal mucosa upon exposure to *L. plantarum *WCFS1, a single colony isolate from NCIMB8826 (National Collection of Industrial and Marine Bacteria, Aberdeen, UK). This strain was originally isolated from human saliva and is used as a model microbe in the study of host-microbe interactions, due to its established taxonomy, its ability to survive gastrointestinal (GI) transit [8] and its putative role as a probiotic species [9]. The availability of the complete genome sequence of *L. plantarum *WCFS1 [10] has strongly facilitated the study of its response to the conditions encountered in the mammalian GI tract.

In the present study, we applied a recently developed *in vivo *intestinal perfusion technique [11] to assess the transcriptional and proteomic response of intestinal mucosa to short (1 h) and extended (6 h) exposures to *L. plantarum *WCFS1 in healthy subjects. These studies focused on the proximal small intestine, because in healthy people, only small amounts of microbes reside in this intestinal region, whereas it encounters and perceives large amounts of food-derived microbes. The fact that only small amounts of microbes reside in that specific niche enables to determine effects of the *L. plantarum *WCFS1 exposures without interference of other mucosa-microbe interactions, as would be the case in the distal small intestine or the colon, which harbour a large microbial community. This manuscript describes, for the first time in healthy subjects, the responses of the intestinal mucosa to intraluminal microbes. Biological interpretation of the gene expression analysis revealed that especially genes associated with lipid metabolism, cellular proliferation, cell death and survival and immune responses were modulated. The impact of the microbes on transcription of genes involved in these processes is presented and discussed in detail.

## Results

The mucosal responses analyzed and presented here represent the acute response of epithelial cells interacting with *L. plantarum *cells and their secreted products and that of cells from non-interacting, neighboring epithelial cells in the lamina propria, which are not directly exposed to luminal contents, and other cell types such as immune cells.

### Study 1

We observed significant changes in 669 gene reporters; 225 genes upregulated and 444 gene reporters downregulated (see Additional file 1). The fold changes of these differentially expressed genes ranged from 0.56 to 1.33. A fold change of 0.56 means that 56% of the expression of the gene in the placebo intervention remains after the *L. plantarum *WCFS1 intervention, whereas a fold change of 1.33 means that the expression of that gene increased by 33% due to the *L. plantarum *WCFS1 exposure.

The biological implications of the altered transcriptional profiles were first identified using the pathway analysis software suite GenMapp. Table 1 shows the 24 pathways, based on the Gene Ontology database, that had a Z-score > 3, and 3 gene reporters or more were differentially expressed. Among these processes, protein biosynthesis and -metabolism, ribosome-associated processes, processes involved in protease activity and fatty acid oxidation seem to be the most important modulated biological processes

**Table 1:** GenMAPP pathways affected by a 1-h exposure of duodenal mucosa to *L. plantarum *WCFS1

**GO Name**	**Changed**	**Measured**	**Total**
caveola	3	4	4
Golgi stack	3	10	12
protein biosynthesis	20	173	252
peptidyl-prolyl cis-trans isomerase activity	6	26	32
protein metabolism	3	13	16
structural constituent of ribosome	18	153	232
isomerase activity	11	77	96
cytosolic large ribosomal subunit (sensu Eukarya)	7	38	39
cytoplasm	35	512	636
ribosome	13	117	167
heterogeneous nuclear ribonucleoprotein complex	4	16	17
translational initiation	3	18	26
endoplasmic reticulum	22	234	271
ER to Golgi transport	4	18	20
GTPase activity	3	21	38
regulation of translational initiation	4	25	32
fatty acid beta-oxidation	3	12	13
ubiquitin conjugating enzyme activity	7	46	75
chymotrypsin activity	10	79	119
catalytic activity	6	96	210
regulation of translation	3	24	27
metabolism	19	243	408
trypsin activity	10	79	134
cytosolic small ribosomal subunit (sensu Eukarya)	5	29	30

Biological processes with relatively high numbers of gene reporters changed, based on the Gene Ontology (GO) database. The table shows the process title (*GO name*), the number of gene reporters changed changed (*changed*), measured with the microarrays (*measured*) and the total number of genes present in the pathway MAPP (*total*) (for detailed description of pathway selection, see Methods).

### Study 2

The 6-h *L. plantarum *challenge significantly modulated the expression of 424 gene reporters; 383 were upregulated and 41 were downregulated (see Additional file 2). The fold changes of these differentially expressed genes ranged from 0.70 to 1.78. Genmapp pathway analysis showed that, based on Gene Ontology databases with the criteria set at Z-score > 5, and 3 gene reporters or more differentially expressed, 22 pathways were mediated by the intervention (Table 2). Processes which are associated with the MHC class I and II and other immune response and antigen processing related processes were modulated by the intervention, as well as several processes which are involved in energy metabolism (mainly fatty acid beta-oxidation, tricarboxylic acid cycle and electron transport chain).

**Table 2:** GenMAPP pathways affected by a 6-h exposure of duodenal mucosa to *L. plantarum *WCFS1

**GO Name**	**Changed**	**Measured**	**Total**
energy pathways	14	83	92
Cytoplasm	26	514	636
oxidoreductase activity	30	318	453
MHC class II receptor activity	5	11	31
tricarboxylic acid cycle	7	22	29
fatty acid beta-oxidation	5	12	13
electron transport	27	221	346
antigen presentation\, exogenous antigen	4	10	30
antigen presentation\, endogenous antigen	4	7	13
antigen processing\, exogenous antigen via MHC class II	4	10	30
antigen processing\, endogenous antigen via MHC class I	4	10	18
endoplasmic reticulum	26	235	271
Mitochondrion	29	365	464
cytochrome-c oxidase activity	5	16	27
Microsome	14	94	118
unspecific monooxygenase activity	6	23	23
lipid metabolism	9	104	129
Glycolysis	8	39	56
Cytosol	12	126	141
mitochondrial membrane	4	10	13
inner membrane	6	26	33
hydrogen ion transporter activity	3	26	33

Biological processes with relatively high numbers of gene reporters changed, based on the Gene Ontology (GO) database. The table shows the process title (*GO name*), the number of gene reporters changed changed (*changed*), measured with the microarrays (*measured*) and the total number of genes present in the pathway MAPP (*total*) (for detailed description of pathway selection, see Methods).

The proteome analyses of the tissue samples obtained in study 2 showed that two proteins differed consistently in all volunteers in response to the 6-h exposure to *L. plantarum *(data not shown). One of these spots was identified as microsomal triglyceride transfer protein (MTP), while the analysis failed to allow identification of the second spot. The microarray results showed that the expression of the gene encoding MTP (probeset 205675_at on the Affymetrix U113A microarray and indicated by UniGene reference Hs.195799) was increased by 19.3%, as a result of the exposure to *L. plantarum*. Paradoxically, the same gene appeared to be down-regulated by 36.8% in the 1 h *L. plantarum *intervention study, but this difference did not reach statistical significance. The tissue samples from study 1 were not subjected to proteome analyses.

### Comparative analysis of study 1 and 2

Fifty-four genes were regulated in both the 1 h and 6 h perfusion microarray datasets. Of these genes, 4 were differentially expressed in the same direction in both studies (3 were upregulated and 1 downregulated), 2 were upregulated and downregulated, and 48 were downregulated and upregulated in study 1 and 2, respectively. Below, the findings of the two studies are compared in detail using the Ingenuity Pathways Analysis (IPA) software tool (see Availability and requirements section for URL). The statistical assessments of significance of representation of the metabolic and signaling pathways that are part of the IPA output are based on a right-tailed Fisher's Exact Test. This test calculates the probability that genes participate in a given pathway, relative to their occurrence in all other pathway annotations covered by Ingenuity.

#### Lipid biosynthesis and fatty acid metabolism

**Figure 1:**
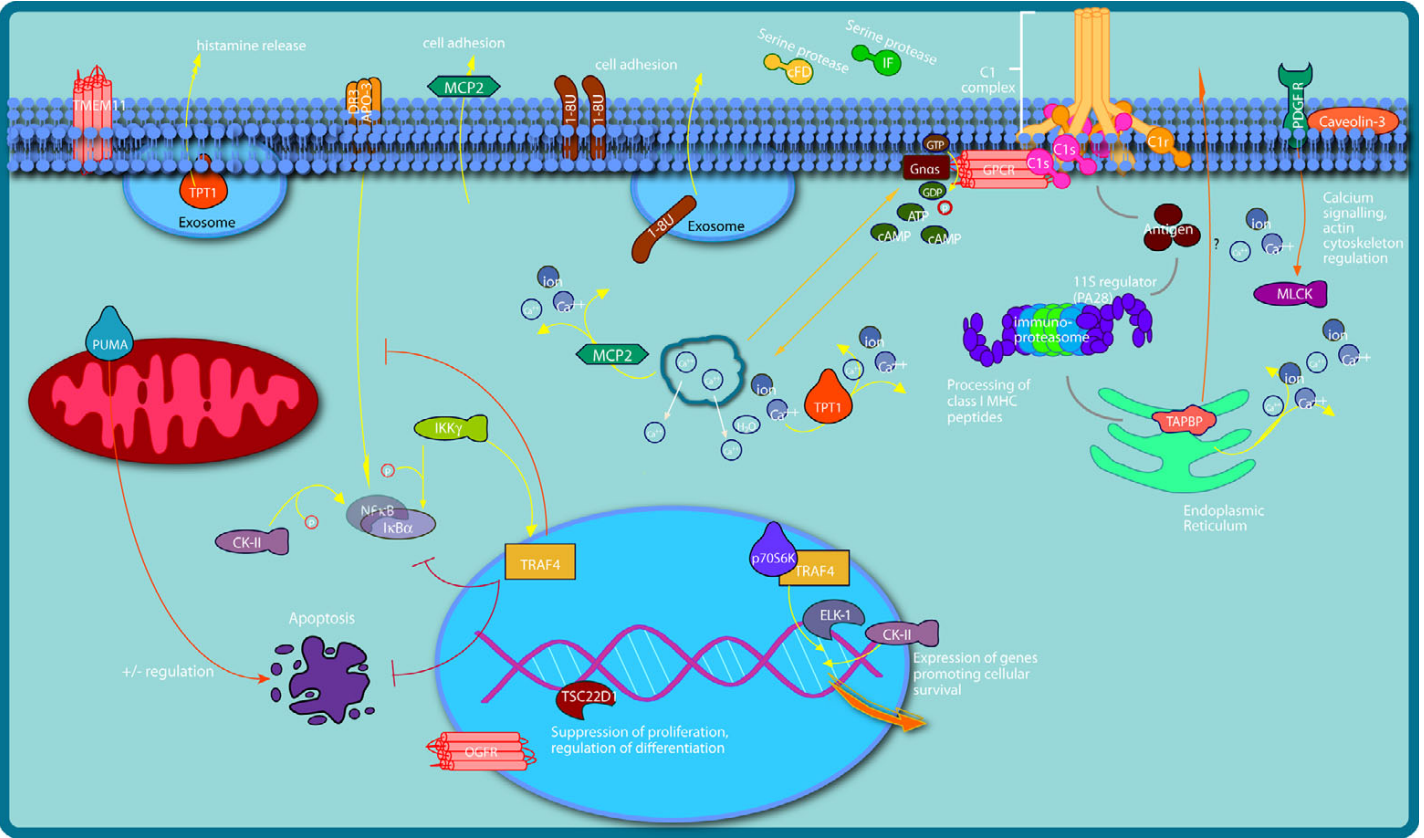
**Cellular responses after one hour exposure to *L. plantarum***. After continuous injection of *L. plantarum *WCFS1 for one hour into the proximal duodenum, proteins that participate in integrated signalling, metabolic and response pathways were modulated in intestinal mucosa. The left area shows genes encoding proteins involved in the regulation of cell death and survival, the central and right areas shows diverse signalling pathways and proteins that are involved in complement activation (immune response; may include genes expressed in immune cells). Note the involvement of nuclear proteins in modulation of cell death and survival.

**Figure 2:**
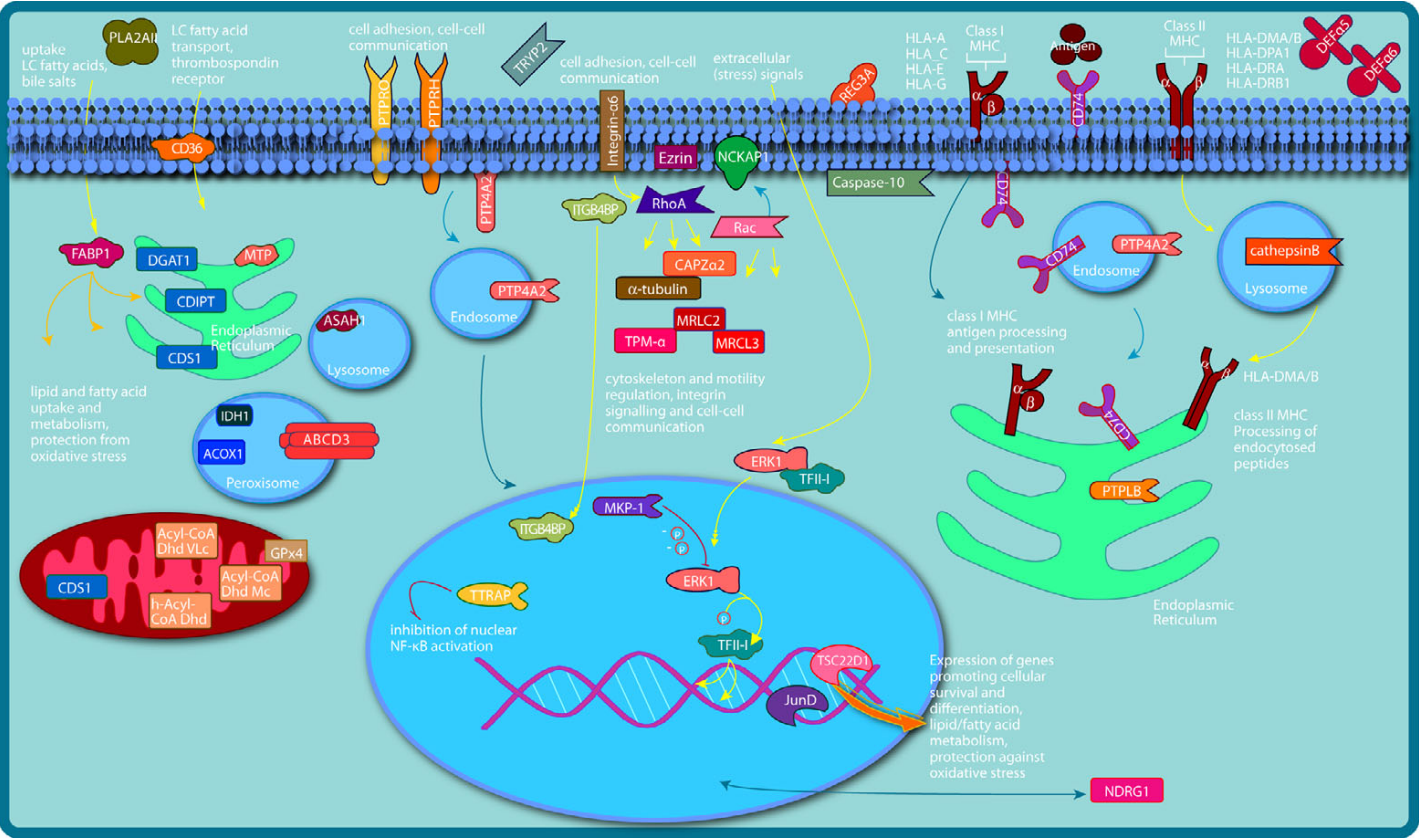
**Cellular responses after six hours exposure to *L. plantarum***. After continuous injection of *L. plantarum *WCFS1 for six hours into the proximal duodenum, proteins involved in fatty acid metabolism, cell motility and immune responses were modulated. Genes encoding proteins involved in fatty acid metabolism are in the upper left area of the image, those involved in cell motility (may include genes expressed in immune cells) are in the central area, and genes involved in innate and adaptive immune response are in the upper right area. Note the modulated ERK/MAPK/MKP-1 signalling pathway that extends into the nucleus.

After 1 h of exposure to *L. plantarum*, three genes involved in lipid and fatty acid metabolism (*ACSL5; CD36 antigen*, and *DEGS1*) were downregulated (Figure 1). After 6 h exposure, the transcription regulators *JUND*, *GTF2I *and *TSC22-D1*, which are involved in protection from oxidative stress and lipid/fatty acid metabolism, *GPX4*, which is involved in protection against oxidative stress, the fatty acid binding protein *FABP1*, the thrombospondin receptor (or fatty acid translocase) *CD36*, six acyl-CoA genes and *isocitrate dehydrogenase-1 *(*IDH1*), which is one of the more "central" hub proteins were all upregulated. However, important central regulatory proteins such as the obesity gene *leptin *or *PPAR-γ *were not differentially expressed after 6 h exposure (Figure 2).

#### Major transcription factors and proteins mediating cellular proliferation

After 1 h exposure to *L. plantarum *WCFS1, four major transcriptional regulators were all found to be downregulated: the oncogene product *MYC *(*c-Myc*), *FOS *(*c-Fos*), *JUN *(*c-Jun*) and the p65 subunit of the NF-κB transcription complex, *RELA*. From these responses, it appears that downregulation of genes modulating cellular metabolism and proliferation is the central mucosal response to the first perception of *L. plantarum*. None of these regulators were differentially expressed after the 6 h exposure.

#### Cell death and death-inducing factors

Several genes encoding proteins which are involved in regulation and modulation of cell death were regulated in both studies. The tumor necrosis factor receptors (TNFRs) *TNFRS1A*, which is a major receptor for TNF-α and a mediator of apoptosis and regulator of inflammation, and *TNFRSF4 *were downregulated after 1 h infusion of *L. plantarum*, whereas the TNF receptor *TNFRSF25 *(*DR3*) was upregulated after the 1-h exposure to *L. plantarum *WCFS1. Expression of the TNF-related proteins *TNFAIP1 *(*B12*), which is a membrane-located ion channel and a direct target of TNF-induced signaling, *transforming growth factor alpha (TGF-α)*, and the TNFR inducer *interleukin 1-β *were downregulated as well after 1 h of exposure to *L. plantarum *WCFS1.

Under cellular immune-response stimulating conditions, translocation of the transcription factor NFκB to the nucleus promotes the expression of predominantly proinflammatory cytokines. The p65 (RelA) component of NF-κB was downregulated after 1 h exposure. Expression of one of the upstream regulators of NF-κB function, *IKBKG *(*IKK-κ *or *NEMO*), was upregulated. IKK phosphorylates IκB, thereby targeting IκB for proteolytic degradation and indirectly enabling nuclear translocation of NFκB. *TNF receptor-associated factor 4 (TRAF4)*, which negatively regulates NF-κB activation upstream from IKK, was also upregulated after 1 h exposure.

In line with the overall down- or nonregulation of death-mediating pathways, one of the non-TNF proteins which belong to the group of direct inducers of cell death by extracellular signals, the Fas-activated serine/threonine kinase *FASTK*, was downregulated after 1 h exposure to *L. plantarum*.

The genes *TPT1 (or histamine releasing factor), cytokine induced apoptosis inhibitor 1 (cIAP-1)*, *mitochondrial PUMA (BBC3), cathepsin B (CTSB), ATP6V0A1, cathepsin L and LAMP2*, all of which are associated with cell-death related responses, were differentially expressed after the 1 h exposure to *L. plantarum *WCFS1. We found that those genes involved in regulation of cell death during acute responses were not regulated or expressed after 6 h exposure and that no other death-regulating genes were expressed as well. Of the TNFR family, only *TTRAP*, the TRAF and TNF receptor-associated protein, was upregulated after 6 h exposure to *L. plantarum*.

#### Immune responses

After 1 h exposure to *L. plantarum*, *interleukin-1 (IL-1), IL-1 receptors IL-1R *and *IL-1-like receptor IL1RL1 (ST2) *were downregulated. The pro-inflammatory cytokine receptor *IL8R1 *was downregulated as well. No regulation of these genes was found after 6 h exposure.

Several genes activating innate immune responses were upregulated after 1 h exposure (Table 3). Among these was IF, the gene encoding the complement factor I, a serine protease which can activate the classical complement pathway of innate immune response. A second, CFD (adipsin) is also a serine protease and activator of the complement pathway. C1S, a third serine protease and activator of complement pathway is involved in activation of G-protein-coupled receptor pathway and contains a calcium-binding EGF domain. C1R is a protein similar to C1S and is also part of complement pathway. All the encoding genes were upregulated after 1-h exposure to *L. plantarum *WCFS1. In line with upregulation of C1S, downregulation of one of its inhibitors, SERPINE2, was observed.

**Table 3:** Immune-related responses of human intestinal mucosal cells to *L. plantarum *WCFS1

component	function	regulation after 1 hr exposure	regulation after 6 hrs exposure
IL-1	interleukin-1 cytokine, induction immune responses		-
IL-1R	receptor for interleukin-1 (IL-1)		-
IF	complement factor I, serine protease, activator of complement pathway of innate immune response		-
CFD (adipsin)	serine protease, activator of complement pathway of innate immune response		-
C1R	serine protease, activator of complement pathway of innate immune response and GPCR ^1 ^pathway		-
C1S	serine protease, activator of complement pathway of innate immune response and GPCR ^1 ^pathway		-
C8G	part of antibacterial membrane attack complex (MAC)		-
CD74	TM receptor, antigen presentation, immune response and humoral defence	-	
CD93	cell-surface glycoprotein, part of complement component 1, activator of macrophage and cellular adhesion		-
HLA-A *	regulation of NK cell cytotoxicity	-	
HLA-C	endogenous antigen presentation	-	
HLA-E	immune response regulation, protection from NK cells	-	
HLA-G	regulatory/suppressive to T cells, cytokine tolerance	-	
HLA-DMA	antigen presentation, detection of microbes, CLIP release	-	
HLA-DMB	antigen presentation, detection of microbes, CLIP release	-	
HLA-DPA1	antigen presentation derived from extracellular proteins	-	
HLA-DRA	antigen presentation derived from extracellular proteins		
HLA-DRB1	antigen presentation derived from extracellular proteins	-	

^1 ^G-protein coupled receptor

* HLA (human leukocyte antigen) are transmembrane receptors that are all part of MHC complex pathway, HLA-x are class I, HLA-Dxxx are class II paralogues.

Green triangles refer to downregulation, and red triangles to upregulation of genes related to immune responses.

The C8G gene which encodes the γ polypeptide of complement component 8 was downregulated in the 1 h exposure study. C8G is part of the antibacterial Membrane Attack Complex (MAC), a cytolytic protein complex involved in damaging bacterial cell membranes. Also CD93 (C1QR1), a cell-surface glycoprotein and part of complement component 1, was downregulated. In contrast to 1 h exposure, none of these genes were regulated after 6 h exposure.

Parts of the MHC class I pathway were found to be regulated after the 1 h exposure. The activating proteasome α and β subunits (PSME1/2) were among the few upregulated genes. The chaperone HSP70 which plays a role during processing of cytosolic antigens before uptake into the ER, was downregulated. Although no regulation of the TAP1 or TAP2 transporters was found, the TAP-binding protein (TAPBP) which mediates the interaction between *de novo *produced MHC class I molecules and the transporter associated with antigen processing (TAP), was upregulated. Only one of the MHC transmembrane (TM) receptors, HLA-DRA was regulated after 1 h exposure. The (downregulated) gene product is expressed in antigen-presenting cells (APCs) and is involved in immune response, exogenous antigen presentation and processing.

After 6 h exposure to *L. plantarum *WCFS1, the intestinal mucosa showed a different immune response-related expression profile. Several HLA-type TM receptors belonging to MHC class I and class II were upregulated (Table 3). The upregulated HLA-A TM receptor is involved in immune responses and modulates, upon interaction with natural killer (NK) cells, cytotoxicity. The HLA-E gene encodes an MHC-class protein with an immunoregulatory function; surface localization of the protein can protect expressing cells from NK cell-mediated lysis. The HLA-DMA TM receptor was among the class II receptors that were upregulated after 6 h exposure to *L. plantarum *WCFS1. This receptor is involved in immune response via antigen presentation and processing and in the detection of microbes. The TM receptor CD74 antigen was also upregulated after 6 h exposure. This receptor is involved in antigen presentation, immune response and humoral defense mechanism, but does also promote cell proliferation and negative regulation of apoptosis.

*Q-PCR *The microarray dataset was verified with real-time quantitative PCR (QPCR). The observed up- or downregulation of eight out of ten genes was confirmed. The Q-PCR analysis of two genes, GUCA2A and PCNA, did not confirm the microarray data. In addition, QPCR analysis of the gene representing MTP confirmed the microarray results.

## Discussion

Human *in vivo *studies aiming to elucidate the interaction between microbes and the intestine are hampered by medical and ethical limitations. Previously, mainly animal models have been employed to investigate mucosal responses to microbes. In germ-free mice, colonization with commensal bacteria induced genes involved in nutrient absorption, mucosal barrier fortification, xenobiotic metabolism, angiogenesis and postnatal intestinal maturation [12], and innate immunity [13]. These studies clearly revealed that the exposure to commensal bacteria modulates the expression of many genes with diverse functions in general gut physiology. It is not clear how these findings relate to the human *in vivo *situation, in which a complex community of different microbes is present. Data on the impact of commensal microbes on the transcriptome of human gut mucosa *in vivo *is restricted to studies describing the effects of supplementation of *L*. GG [7] and of *Bacillus clausii *[14] on intestinal transcriptional responses in oesophagitis patients. In the present study, the impact of *L. plantarum *WCFS1 on gene transcription and pathway regulation was investigated in a human *in vivo *model in healthy volunteers that enabled to administer the microbes directly into the small intestine and sample intestinal tissue for transcriptional profiling. To the best of our knowledge this is the first example of a study that employs post-genomic technologies to unravel specific host-microbe interactions in healthy human subjects. In two subsequent intervention studies, effects of 1- and 6-h continuous injection of *L. plantarum *WCFS1 were investigated. An exposure time of 1-h was chosen as the 'short term' exposure time, whereas an exposure time of 6 h was considered as a prolonged exposure. Previous studies in our lab showed that after a 6-h period, problems may arise due to the long fasting period, during which subjects remain at rest and, hence, this was considered as the maximal time frame for the chosen experimental set-up.

The two studies presented here showed regulation of several hundreds of genes in small intestinal mucosa induced by intraluminal ingestion of *L. plantarum*. Exposure of proximal small intestinal mucosa for 1 h to *L. plantarum *provoked a transcriptional response, which at least in part appeared to be modified in the prolonged (6 h) exposure. The temporal changes in gene transcription during the first hours of intraluminal injection of *L. plantarum *as described in this manuscript provides an in-depth overview of the cellular host response to intraluminal non-pathogenic microbes. The observed counter-regulatory responses are important for the maintenance of normal gut homeostasis. This was confirmed by the observations of the proteomics assay in the prolonged exposure study, in which only two proteins were consistently found to be differentially present after the *L. plantarum *exposure compared to placebo. In the acute exposure study, no attempts were undertaken to investigate differences in protein profiles, because the exposure period of 1 h was expected to be too short to provoke substantial differences at the protein level.

Lipid and fatty acid uptake and metabolism participate in a diversity of cellular functions including membrane biogenesis, general metabolism and (bacterial) signaling. With increasing mucosal exposure time to *L. plantarum*, the transcriptional profile of lipid and fatty acid uptake and metabolism was also affected, reflecting an increase of fatty acid and lipid metabolism.

After 1 h perfusion, several genes involved in lipid and fatty acid metabolism or uptake and transport (ACSL5; CD36 antigen and DEGS1) were downregulated, suggesting that the mucosal cells did not actively take up or metabolize additional fatty acids and lipids during the interaction with *L. plantarum *in comparison to placebo-treated mucosa. In contrast, after the prolonged exposure of 6 h, three transcription regulators involved in lipid/fatty acid metabolism and protection from oxidative stress as a result of this metabolism (JUND, GTF21 and TSC22-D1) were upregulated in comparison to the placebo treatment (Figure 2). One important gene involved in protection against oxidative stress, glutathione peroxidase (GPX4), was also upregulated after 6-h exposure to *L. plantarum*.

The CD36 glycoprotein, which plays a role in cellular adhesion and in the regulation of fatty acid transport and uptake, and participates in cellular control of oxidative stress by binding to oxidized low-density lipoprotein [15], was downregulated after 1-h exposure, but was upregulated after 6 h exposure to *L. plantarum*. The fatty acid binding protein FABP1 was upregulated in the 6-h exposure study, as was the microsomal and ER-localized triglyceride transfer protein, MTP, which is discussed in more detail below. Hence, it is clear that the prolonged exposure to *L. plantarum *induced upregulation of genes involved in fatty acid uptake. Several genes participating in mitochondrial and peroxisomal fatty acid metabolism (e.g. acyl-CoA genes) or membrane sphingolipid metabolism (ASAH1) were upregulated as well (Figure 2).

Microsomal triglyceride transfer protein (MTP) was modulated on the transcriptional and on the protein level during the interaction with *L. plantarum *in the 6-h exposure study. MTP is involved in intracellular lipid transport and secretion, and is primarily expressed in liver and intestinal tissues [16]. Recently, MTP was also found to be involved in modulating the intestinal immune response to antigens by regulating type I CD1 molecules [17,18], which are major histocompatibility complex (MHC) class I-homologues. It was also shown that MTP affects antigen presentation and is able to regulate CD1a, CD1b and CD1c production, hence supporting the idea that MTP is important in the host response to microbial pathogens. This is in agreement with the other MHC-related responses observed in this study, as described below. Overall, mediation of MTP in response to extra- and intracellular signals may affect the mucosal response to pathogens, and may also affect lipid metabolism and plasma lipoprotein levels. MTP inhibitors may be used to promote weight loss [19], but they may have detrimental side effects, such as fat accumulation in the liver and small intestine. The observation that MTP was non-significantly downregulated in the 1-h exposure study, whereas it was upregulated in the extended, 6-h exposure study, presents a challenging situation to speculate on the eventual physiological consequences. The role of MTP in lipid metabolism, a process that was affected by *L. plantarum*, and its role in the immune response involving dendritic cells [18] could suggest that upon *L. plantarum *exposure, a mucosal response was triggered that initiated microbe sensing by dendritic cells, which was at least in part mediated by MTP, and stimulated lipid metabolism-related processes in intestinal mucosa.

The down-regulation of major transcription factors stimulating cell proliferation observed in the acute exposure study suggests that cell proliferation is suppressed after the first perception of *L. plantarum *cells. The initial down-regulation of the cellular cycle may be the result of a shift in cellular activity towards microbial perception, cell defense and immune responses. Indeed, both during acute (1 h exposure) and later responses, modulation of immune response pathways and cellular death, survival and proliferation was observed.

Transforming growth factor-β (TGF-β) is an important regulator of cell proliferation [20]. Although no regulation of TGF-β was observed in the datasets, the TGF-β-early-response genes TSC22 displayed exposure-time dependent differential regulation in time, with downregulation after 1 h (TSC-22D1, -3) and upregulation after 6 h (TSC-22-D1). TSC-22 is an important downstream component of TGF-β and PPARγ signaling during intestinal epithelial cell differentiation. Both TGF-β and PPARγ signaling pathways are important for inhibiting excessive epithelial cell proliferation in the intestine [21]. The downregulation of proliferation in the acute phase, after the 1-hr exposure to *L. plantarum*, appeared to be further enhanced by an upregulation of the proliferation-inhibiting gene product IFITM3. Generally, proliferation rates are primarily determined by two main and interacting pathways, i.e., the NFκB and the mitogen-activated protein (MAP) kinase pathways. After 6 h exposure, the MAP kinase ERK1 (MAPK3) was upregulated. In the cellular context, ERK1 may have acted as an inhibitor of cellular over-proliferation by downregulating important oncogene regulators such as c-Fos and by antagonistic effects on ERK2 function, a related MAPK, that mediates proliferative signals [22].

NFκB is one of the key regulators of both cell proliferation and apoptosis [23]. Remarkably, after 1 h treatment with *L. plantarum*, only down-regulation of the p65 subunit of NFκB was found, while no component of NFκB was differentially expressed after 6 h exposure. The exact role of NFκB in cell proliferation is not clear, but it was shown that NFκB is required for cell proliferation by mediating the expression of c-Myc [24], which stimulates the transcription of genes with an important role in cell cycle control, like cyclins, CDKs and E2F [25].

NFκB plays an important role in the regulation of apoptosis. It suppresses apoptosis by inducing the expression of several anti-apoptotic proteins and by modulating the expression or activity of pro-apoptotic proteins [25]. Paradoxically, NFκB also exerts pro-apoptotic activity [26]. NFκB itself exists as an inactive cytoplasmic complex, bound to inhibitory proteins of the IκB family. Upon stimulation, TRAFs recruit NFκB -inducible kinases (NIK) that phosphorylate the IKK complex that subsequently triggers ubiquitination of the IκB, which makes it possible for the NFκB complex to translocate from the cytoplasm to the nucleus, where it activates transcription of various genes [27]. One caspase recruitment domain protein, CARD10, induces NFκB activity through interactions with the IKK complex [28]. This CARD10, was upregulated after 6 h exposure, suggesting NFκB activity induction. However, the simultaneous upregulation of TTRAP, a gene that encodes a protein that inhibits NFκB by binding to TNF receptors [27] may have antagonized the presumptive CARD10 induction of NFκB complex.

Cell death induction during interactions with microbes occurs primarily through TLR signaling and expression of death-inducing cytokines, such as tumor necrosis factor (TNF) or Fas ligand (FasL) with concomitant increased expression of the appropriate death-signaling receptors. One receptor of the TNF family, TNFRSF25, was upregulated during acute response (1 h exposure). TNFRSF25, also called death receptor 3 (DR3), induces NFκB activation and thereby mediates the activation of apoptosis [29].

Although several genes associated with cell death were found to be differentially expressed, none of these are known to stimulate or execute cell death. Importantly, with progressing duration of exposure to the microbes, cells did not appear to maintain the cellular "gestation" phase with downregulated metabolism that characterized the acute 1-h response to *L. plantarum *but rather, to switch to a more proliferative phase with an overall expression profile characterized by upregulation of genes involved in cellular growth, proliferation and development. Notwithstanding this shift to cellular proliferation, monitoring of bacterial presence was still evident, as can be deduced from the upregulated expression of antimicrobial α-defensins and the major histocompatibility complex (MHC) antigen processing and presentation pathway.

Important MHC and other innate immune system-related receptors and effectors were differentially expressed after 1 and 6 h exposure, respectively. After the 1 h exposure to *L. plantarum*, genes encoding major complement component 1 proteins were upregulated, representing a group of proteins that mediate the "classical pathway" of the complement immune response. In contrast, the C8G gene, which encodes the γ polypeptide of complement component 8 was downregulated. This gene is part of the antibacterial Membrane Attack Complex (MAC), a cytolytic protein complex involved in damaging bacterial cell membranes. The observation that this antibacterial protein was down-regulated might imply that the immune system in the intestinal mucosa can perceive *L. plantarum *within 1 h and recognize these as commensal and non-pathogenic bacteria.

Prolonged infusion of *L. plantarum *induced MHC antigen processing and presentation pathways indicating that mucosal epithelia actively performed a bacterial monitoring and identification process. CD74, one of the major receptors, which can be bound by bacteria, was one of the upregulated genes. This receptor is involved in antigen presentation and immune responses [30], but does also promote cell proliferation and negative regulation of apoptosis.

Several MHC-I and MHC-II transmembrane receptors were upregulated after the 6 h exposure. After prolonged exposure to lactic acid bacteria, CD74 and HLA-DMA and -B were upregulated. These receptors participate in the MHCII pathway of antigen endocytosis, processing and presentation [31,32]. The increased expression of HLA-E indicates that epithelial cells were actively protected from potentially damaging immune system responses. Increasing the amount of HLA-E present at the cell surface renders cells less sensitive to natural killer-cell-mediated lysis. Stabilization of HLA-E expression is among others mediated by HLA-A2 [33] and the expression of this protein is also upregulated after 6 h.

Summarizing, complement 1 was induced during acute responses and MHC antigen processing and presentation pathways were being expressed after 6 h exposure, which suggests the activation of bacterial monitoring and identification process. Based on the microarray data, no inflammatory immune responses were executed. Rather, the expression profile after 6 h of exposure to *L. plantarum *is in agreement with cellular proliferation. However, it should be noted that after 6 h perfusion, the Paneth-cell-specific defensin α5 was upregulated, which indicates that the luminal microbes were perceived and their numbers controlled by immune responses from the crypt regions of the intestinal mucosa.

The Q-PCR analyses were in agreement with the microarray data. The gene expression results of eight out of ten genes were confirmed, which is in line with the consensus that the Affymetrix microarray platform provides a reliable platform to measure gene expression [34]. The observed differences between these two technologies are in line with the observations of others [34], and are mostly caused by differences in probe sequence and thus target location.

The present manuscript provides an in-depth overview of the initial transcriptional response of healthy intestinal mucosa upon its interaction with live bacterial cells of *L. plantarum*, describing time-dependent changes in transcriptional responses after 1 and 6 h of bacterial exposure to *L. plantarum*. The observed changes were modest, as genes were affected by maximally 78%. This is in concordance with the impact of an intervention study by our group in humans, in which also only modest impacts on fold changes, up to doubling of the gene transcripts, was observed [11]. The biological observations of the present study were in line with those observed in previous studies on the effects of a 30-d supplementation with the probiotic microorganisms *L. rhamnosus *GG and of *Bacillus clausii *on transcriptional responses of small intestinal mucosa in humans [7,14]. Immune modulation, cell growth, and cell-cell signaling were mediated at the transcriptome level by all three microbes (*B. clausii, L. rhamnosus *GG and *L. plantarum *WCFS1). In addition, our study revealed that *L. plantarum *WCFS1 mediates fatty acid uptake- and metabolism, contributing not only to lipid metabolism but also to immune response modulation as discussed above. It should be noted that, although the authors of the 30-d supplementation studies state that effects of probiotics on healthy duodenum were investigated, the use of medication (proton pump inhibitors) as well as the small sample size (n = 3 in each group) hamper the generalization of their results and, hence, comparison with the results of the present study may not be justified. We would like to emphasize that the effects on gene transcription, observed in the present study, may not be specific for *L. plantarum *WCFS1. As indicated above, some biological effects were also induced by other microbes. Future studies should reveal whether *L. plantarum *WCFS1 induces specific and unique responses in the gastrointestinal tract.

In conclusion, the present study showed that *L. plantarum *WCFS1 induced time-dependent transcriptional changes in intestinal mucosa in healthy subjects. Among a variety of biological processes, especially lipid metabolism, cellular proliferation, cell death and survival and immune responses were modulated. The gene expression profiles suggest that cell death and pro-inflammatory responses were triggered, but not executed. This extensive exploration of the human response to *L. plantarum *WCFS1 may eventually provide molecular support for specific or probiotic activity of this strain and/or species, and exemplifies the strength of the applied technology for the identification of the potential bio-activity of microbes in the human intestine.

## Methods

This study encompassed two human intervention studies, which are described below under 'study 1' and 'study 2', respectively. Both studies were approved by the University Hospital Maastricht Ethical Committee, and conducted in full accordance with the principles of the 'Declaration of Helsinki' (52^nd ^WMA General Assembly, Edinburgh, Scotland, Oct 2000). All subjects gave their written informed consent prior to their inclusion into the study.

### Preparation of L. plantarum WCFS1

*L. plantarum *WCFS1 was grown on MRS medium under anaerobic conditions, following standard laboratory procedures. Fifteen minutes prior to each experiment with *L. plantarum *WCFS1, 10^11 ^freshly prepared *L. plantarum *WCFS1 were resuspended in 600 mL physiological saline solution, containing 10 g/L glucose, at 37°C.

#### Study 1

##### Subjects

Eight healthy non-smoking volunteers (24 ± 4 y) without a history of GI symptoms and free of any medication, were investigated on two separate occasions in a randomized placebo-controlled crossover study.

##### Protocol

After an overnight fast, mucosal tissue samples were obtained from the horizontal part of the duodenum, approximately 15 cm distal to the pylorus, by standard flexible gastroduodenoscopy and a perfusion catheter was placed orogastrically in the proximal small intestine, as described previously [11]. Briefly, a catheter that enabled to inject a fluid 5 cm distal to the pylorus was inserted during a gastroduodenoscopy procedure. Catheter positioning was performed under short interval fluoroscopic control. No sedatives were given to the volunteers. Subsequently, during the first 180 min of the experiment, a physiological saline solution containing 10 g/L glucose was infused continuously at 10 ml/min using a peristaltic pump. After this period, which allowed establishment of steady state conditions in intestinal fluid fluxes, physiological saline containing 10 g/L glucose with or without, in total, 1 × 10^11 ^*L. plantarum *WCFS1 was infused for one hour. Fluids were maintained at 37°C using a shaking water bath. Subjects remained in the supine position until the end of the experiment, and food or beverage consumption was not allowed during the experiment. After the perfusion experiment, the perfusion catheter was removed by gently pulling out the catheter. A second gastroduodenoscopy was performed exactly 15 minutes after the perfusion experiment to obtain tissue samples from the same intestinal region as earlier that day. The entire protocol was repeated on another day, 13 to 16 days after the first experiment, to randomly investigate the effects of placebo or *L. plantarum *WCFS1. In all tissue samples, gene expression levels were measured using genome-wide microarrays (Affymetrix U133A; see below). No samples were taken for proteome analysis purposes, because changes at the protein level were not expected to occur within the 1-h exposure time to *L. plantarum *WCFS1.

#### Study 2

##### Subjects

Seven healthy non-smoking volunteers (28 ± 6 y), without a history of gastrointestinal complaints and free of any medication, participated in a randomized placebo-controlled crossover study.

##### Protocol

After an overnight fast, an intraduodenal feeding catheter (naso-intestinal tube, Flocare^® ^Bengmark, Nutricia Healthcare S.A., Chatel-St. Denis, Switzerland) was placed nasogastrically following the manufacturers instructions. No sedatives were given to the volunteers. After positioning of the catheter in the small intestine (tube tip positioned 5–10 cm distal to the pylorus), a physiological saline solution containing 10 g/L glucose and a total of 10^12 ^*L. plantarum *WCFS1 or, randomly on another test day, only physiological saline solution containing 10 g/L glucose, was injected continuously at 6.7 ml/min for 6 h. The concentration of bacteria present in the fluid was the same in both studies, which was important in order to allow proper comparisons between the different studies. The infusion rate was less than that in study 1, to avoid injecting too large amounts of fluid and bacteria, which could have induced gastrointestinal symptoms. Fluids were maintained at 37°C using a shaking water bath. Subjects remained in the supine position until the end of the experiment, and food or beverage consumption was not allowed during the experiment. After this 6-h period, tissue samples were obtained from the horizontal part of the duodenum, approximately 15 cm distal to the pylorus, by standard flexible gastroduodenoscopy, at approximately 15 cm distal to the pylorus. In all tissue samples, gene expression levels were measured using genome-wide microarrays (Affymetrix U133A; see below). In duplicate tissue samples, differential proteome analyses were performed using the CyDIGE method (2D gel-electrophoresis with minimal fluorescent labeling) with Maldi-TOF MS (see below).

#### Microarray analyses

*RNA isolation *Total RNA was isolated using a TRIzol lysis assay. Next, total RNA of each separate tissue sample was hybridized onto GeneChip microarrays (HG U133A; Affymetrix Inc, Santa Clara, Ca, USA) according to the manufacturer's instructions. Hence, for study 1, 32 RNA samples were hybridized (tissue samples from 8 subjects, obtained before and after the intervention with *L. plantarum *WCFS1 and placebo, respectively) and for study 2, 14 RNA samples (obtained from 7 subjects, taken after to exposure to *L. plantarum *WCFS1) were hybridized onto the chips. Briefly, 1 mL TRIzol (Invitrogen Life Technologies b.v., Breda, The Netherlands) and 10 μL β-mercaptoethanol (VWR International b.v., Amsterdam, The Netherlands) were added to each frozen tissue sample and shaken with a minibeadbeater for 30 seconds. 200 μL Chloroform (Sigma Aldrich Chemie b.v., Zwijndrecht, The Netherlands) was added and the samples were incubated for 3 minutes, followed by phase separation using centrifugation at 21000 g for 15 minutes. The upper aqueous phase was taken and 500 μL 70% Ethanol was added. Subsequently, the extracted RNA was purified using the RNeasy Mini Kit (Qiagen Benelux b.v., Venlo, The Netherlands) with extra DNA digestion by on-column RNase-Free DNase treatment (Qiagen Benelux b.v., Venlo, The Netherlands). RNA purity was determined using nanodrop equipment (ND-1000 spectrophotometer, Isogen Life Science B.V., IJsselstein, The Netherlands). Only RNA samples with a 260/280 ratio between 1.9 and 2.1 were considered for further analysis. RNA integrity was determined using Bioanalyzer technology (Bioanalyzer 2100, Agilent, Santa Clara, USA). RNA samples with RNA integrity numbers (RIN) above 8.0 were considered for further analysis.

### Microarrays

Total RNA was hybridized onto GeneChip microarrays (Affymetrix Inc, Santa Clara, Ca, USA) according to the manufacturers' instructions. For this analysis, five micrograms of total RNA from each sample, and the one-cycle labeling system were applied as recommended by the manufacturer (Affymetrix Inc, Santa Clara, Ca, USA). Microarrays were scanned using a GeneChip Instrument System (Affymetrix Inc, Santa Clara, Ca, USA). The microarray data calculations to obtain detailed information on differentially expressed genes are described in the supplementary material (see Additional file 3).

#### Pathway analysis

The genes analyzed and fold changes were loaded into GenMapp [35] and MAPPFinder [36] and into the GOurmet and Ingenuity Pathway Analysis IPA: (see Availability and requirements section for URL)software packages to evaluate the transcripts in relation to known biological processes, molecular function and cellular component based on Gene Ontology (GO) terms and local maps. Only gene transcripts with either their average intensities for the placebo and treated groups above 500 or average intensities for one of these groups above 1000 and a 10 percent up or down regulation, respectively, were used to obtain a ranked list of pathways with differentially expressed genes.

MappFinder software was used to select the MAPPs with relatively high numbers of differentially expressed genes, which were affected by *Lactobacillus plantarum *WCFS1 compared to placebo. The ranking of regulated pathways was indicated by the individual Z-scores. The Z-score increases if higher numbers of changing genes are found, taking into account the number of genes present in the MAPP that are represented on the array, and the total number of genes involved in the concerning MAPP. MAPPs were selected for further study if the group results (*Lacobacillus plantarum *WCFS1 compared to placebo) reached an arbitrary Z-score of at least 3 on that MAPP for study 1 and at least 5 for study 2, and at least 3 genes were differentially expressed in that pathway. Different Z-score cut-off points were used for the two studies because effects in study 2 were more pronounced and revealed regulation of more biological pathways, thus allowing the use of a more stringent statistical cut-off in the pathway analysis.

### Pathway visualization

Further refinement and biological interpretation of reconstructed pathways was performed by comparison with published information and pathways available from NCBI Entrez Gene info (see Availability and requirements section for URL), the Protein, Signaling, Transcriptional Interactions & Inflammation Networks Gateway database pSTIING, (see Availability and requirements section for URL) and BioCarta charts (see Availability and requirements section for URL). Based on the combined information from IPA and pSTIING and published data, a graphical representation of the microarray results in terms of their encoded and interacting proteins and pathways was reconstructed using the image ("toolbox") palette provided by Biocarta.

*Q-PCR *First Strand cDNA was synthesized using the iScript cDNA Synthesis kit (Bio-Rad, Veenendaal, The Netherlands) according to the manufacturer's instructions. 250 and 500 ng total RNA was used as template for the cDNA reaction of Study 1 en Study 2 respectively. The difference in starting quantities was the consequence of the smaller amount of RNA available for study 1, which was due to a smaller size of the tissue samples obtained in this study. The cDNA was diluted with RNase free H_2_O to a concentration of 5 and 10 ng/μl respectively. IQ Sybr Green Supermix (Bio-Rad, The Netherlands) was used for the Q-PCR. Each Q-PCR reaction of the Q-PCR contained 12,5 μl iQ Sybr Green Supermix, 1 μl of 10 μM gene-specific forward and reverse primers, 2 μl cDNA template solution and 8,5 μl sterile water. The following primers were used. Housekeeping genes were 18SrRNA: gtaacccgttgaaccccatt, ccatccaatcggtagtagcg; GAPDH: tgcaccaccaactgcttagc, ggcatggactgtggtcatgag and Calnexin: ccactgctcctccttcatctcc, cggtatcgtctttcttggctttgg. Genes study 1; GUCA2A: gggttgggaaactcaggaactttg, tacaggcagcgtaggcacag; PCNA: gccactccactctcttcaacgg, tggtgacagaaaagacttcagtatatgc; CD36: ggaatctgtcctattgggaaagtcactgc, ctgggttttcaactggagaggcaaagg; FOS: ctgtgtctcttttctctttctccttagtc, tccagcaccaggttaattccaataatg; DUSP1: agcagaggcgaagcatcatc, acggtggtggtggaggtg. Genes study 2; PLA2G2A: gcagaagtcaactgtgtgagtgtg, gggagggagggtatgagagagg; DEFA6: ggctcaacaagggctttcac, gtatgggacacacgacagtttc; CDS1: tggattcattgctgcctatgtgttatc, ctttagaaagggtggaagtgagtaagtc; REG3A: gctgtcccaaaggctccaagg, atcacatcactgctactccactcc; CD24: gtatttgggaagtgaagactggaagc, agtgttctaaatgtggctattctgatcc. Gene study 1 and 2; MTP: ggacctagcacagaggaatcag, ccaaatccaccagtttcttgaagc. Reactions were run on the My IQ Single Color Real Time PCR Detection System (Bio-Rad, Veenendaal, The Netherlands). The cycling conditions comprised 3 minutes at 95°C and 40 cycles at 95°C for 10 seconds and 60°C for 45 seconds followed by a melting program. The CT values were normalized using the IQ5 Optical System Software version 2.0 (Bio-Rad, Veenendaal, The Netherlands). Gene-transcripts were analyzed using a multivariate Gaussian linear regression, similar to the microarray analysis, with the difference of having the sample concentration, the test day, the perfusion procedure, repeats, and the best housekeeping gene among 18S ribosomal RNA, GAPDH and calnexin included. Detailes of the Q-PCR calculations are provided in Additional file 3.

#### Proteomics analysis

Intestinal biopsies were prepared for protein analysis by sonication in a lysis buffer, and subjected to the 2-D Clean Up kit (Amersham Biosciences, Freiburg, Germany) to remove non-protein material. Protein concentrations were measured using the 2-D Quant Kit (Amersham Biosciences, Freiburg, Germany). The samples were further processed for two-dimensional fluorescence difference gel electrophoresis with the Ettan™ DIGE technique, following the manufacturers instructions [37]. Protein mixtures were separated by 2D electrophoresis, according to their iso-electric points, using the Ettan IPGphor 3 isoelectric Focusing System, and to their molecular weights using an EttanDalt12 electrophoresis system. Samples from the placebo intervention and from the *L. plantarum *intervention were loaded on one IPG-strip, together with a pooled internal standard. After the 2D-electrophoresis, the gels were scanned with a Typhoon confocal laser scanner (Typhoon 9410 Variable Mode Imager, Amersham Biosciences), and the scanned images were loaded into DeCyder software (Decyder 2D, Amersham Biosciences, Freiburg, Germany) to analyse the differences in protein profiles. Differentially expressed protein spots were picked with the Ettan Spot picker (Amersham Biosciences, Freiburg, Germany). Excised spots were subjected to mass-fingerprint identification using Maldi-TOF MS analysis as described elsewhere [38].

## Availability and requirements

Microarray data are available in the ArrayExpress database  under the experiment accession number E-MEXP-1328.

IPA: 

NCBI Entrez Gene info: 

pSTIING: 

BioCarta charts: 

## Authors' contributions

FT conceived of the study, prepared and carried out the human experiments, and drafted the manuscript. PVB performed the bioinformatical- and pathway analyses, and contributed to the draft manuscript. PL was involved in the design of the study and performed the statistical analyses. AK carried out the RNA isolations and the Q-PCR analyses. WDV participated in the design of te study, and helped to draft the manuscript. MK conceived of the study, participated in its design and coordination and helped to draft the manuscript. RJB conceived of the study, participated in its design and coordination, performed the gastroduodenoscopy procedures, and helped to draft the manuscript. All authors read and approved the final manuscript.

## Supplementary Material

Additional file 1 *Genes study 1*. Genes regulated by a 1-h exposure time to *L. plantarum *WCFS1. 669 Gene reporters were differentially expressed by the intervention; 225 genes were upregulated and 444 gene reporters were downregulated by *L. plantarum *WCFS1. Click here for file

Additional file 2 *Genes study 2*. Genes regulated by a 6-h exposure time to *L. plantarum *WCFS1. 424 Gene reporters were differentially expressed by the intervention; 383 genes were upregulated and 41 gene reporters were downregulated by *L. plantarum *WCFS1. Click here for file

Additional file 3 *Microarray and QPCR data calculations*. Detailed description of the calculations of the microarray data and the quantitative RT-PCR analysis. Click here for file
