# Supplementary material for: Identification of the transcriptional response of human intestinal mucosa to Lactobacillus plantarum WCFS1 in vivo
Source: BMC Genomics. 2008 Aug 5;9:374. doi: 10.1186/1471-2164-9-374 (PMC2519092; doi:10.1186/1471-2164-9-374)
Supplement: Additional file 3 — Microarray and QPCR data calculations. Detailed description of the calculations of the microarray data and the quantitative RT-PCR analysis. [file 1471-2164-9-374-S3.doc]

*Microarray data calculations* For study 1, the genes were analyzed using a multivariate Gaussian linear regression [1] comprising the hybridization and labeling spikes, the test day, and the perfusion procedure to enable correction for effects induced by the perfusion technique. For study 2, the genes were analyzed using a multivariate Gaussian linear regression including the hybridization and labeling spikes. The inference criterion used for comparing the models is their ability to predict the observed data, i.e. models are compared directly through their minimized minus log-likelihood. If the numbers of parameters in models differed, they were penalized by adding the number of estimated parameters, a form of the Akaike information criterion (AIC) [2]. For each gene of study 1, the treatment was then accounted for in the model by adding the interaction between the test day and perfusion procedure. For each gene of study 2, the treatment group was then added to the model. The gene under consideration was considered to be differentially expressed if the AIC decreased compared to the model not containing the treatment effect.

All gene-transcripts were analysed using a multivariate Gaussian linear regression ( where is the mean, is the covariance matrix , is the variance, and as both the extra component of variance across subjects and the common covariance among responses on the same subject) including the hybridization and labeling spikes, and a random effect for Perfusion 2, and additionally the test day (placebo or *Lacobacillus plantarum*) and the perfusion procedure (before or after the procedure) for Perfusion 1. The inference criterion used for comparing the models is their ability to predict the observed data, i.e. models are compared directly through their minimized minus log-likelihood. When the numbers of parameters in models differ, they are penalized by adding the number of estimated parameters, a form of the Akaike information criterion (AIC) [2].

For each gene of Perfusion 1, a model containing the relevant covariates mentioned above () was fitted in order to obtain a reference AIC. Then a model containing the treatment effect was fitted by adding the interaction between the test day and the perfusion procedure (Perfusion) (). For each gene of Perfusion 2, a model containing the relevant covariates mentioned above () was fitted in order to obtain a reference AIC. Then a model containing the treatment group (Grp) was fitted (). The gene under consideration was found to be differentially expressed if the AIC of this second model decreased compared to the model not containing the treatment.

*Q-PCR calculations*

Study 1. Gene-transcripts were analyzed using a multivariate Gaussian linear regression, similar to the microarray analysis as described above, with the difference of having the sample concentration, the test day, the perfusion procedure, repeats, and the best housekeeping gene among 18S ribosomal RNA, GAPDH and calnexin included. For each gene, the treatment was then accounted for in the model by adding the interaction between the test day and perfusion procedure. The Akaike information criterion was also used to asses whether there was a treatment effect. Hence, the gene under consideration was found to be differentially expressed if the AIC decreased compared to the model not containing the interaction.

Study 2. Gene-transcripts were analyzed using a multivariate Gaussian linear regression, similar to the microarray analysis, with the difference of having repeats, 18S ribosomal RNA and the best housekeeping gene among 18S ribosomal RNA, GAPDH and calnexin included. For each gene, the treatment group was then added to the model. The Akaike information criterion was also used to asses whether there was a treatment effect. Hence, the gene under consideration was found to be differentially expressed if the AIC decreased compared to the model not containing the treatment difference.

**References**

1. Ewens WJ, G.R. G: **Statistical Methods in Bioinformatics: An**

**introduction**. New York; 2001.

2. Akaike H: **Information theory and an extension of the maximum likelihood principle**. In: *Second International Symposium on Inference Theory: 1973; Akademiai Kiado*

*Budapest*; 1973: 267-281.
